# Importance of ICPMS for speciation analysis is changing: future trends for targeted and non-targeted element speciation analysis

**DOI:** 10.1007/s00216-017-0502-8

**Published:** 2017-07-22

**Authors:** Joerg Feldmann, Andrea Raab, Eva M. Krupp

**Affiliations:** Trace Element Speciation Laboratory, Department of Chemistry, University of Aberdeen, Meston Walk, Aberdeen, AB24 3UE UK

**Keywords:** Inorganic compounds/trace inorganic compounds, Speciation, Mass spectrometry/inductively coupled plasma mass spectrometry, High-performance liquid chromatography

## Abstract

This article is aimed at researchers interested in organic molecules which contain a heteroatom but who have never considered using inductively coupled plasma mass spectrometry (ICPMS) or who have used ICPMS for years and developed numerous methods for analysis of target elemental species. We try to illustrate (1) that ICPMS has been very useful for speciation analysis of metal(loid) target species and that there is now a trend to replace the costly detector with cheaper detection systems for routine target analysis, and (2) that ICPMS has been used and will be used even more in the future for non-targeted analysis of elements which are not normally associated with ICPMS analysis, such as non-metals such as sulfur, phosphorus, chlorine and fluorine.

**Graphical Abstract:**
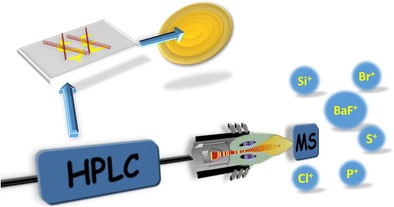
Starting with HPLC-ICPMS for non-targeted analysis of heteroatom containing molecules, once target molecule is identified alternative detectors can be used for routine measurements

Starting with HPLC-ICPMS for non-targeted analysis of heteroatom containing molecules, once target molecule is identified alternative detectors can be used for routine measurements

## Introduction

What is speciation? This has been clarified in the IUPAC definition: the speciation of an element means the distribution of an element amongst defined chemical species in a system. Chemical species are defined as chemical elements: the specific form of an element is defined as to isotopic composition, electronic or oxidation state, and/or complex or molecular structure [1]. However, the IUPAC definition is generally not accepted by different communities [2]. Whereas the analytical community using electrochemical methods defines the speciation of elements as different redox states and stable and labile element species, the community using X-ray absorption near-edge spectroscopy/extended X-ray absorption fine structure spectroscopy or X-ray photoelectron spectroscopy divides the different elemental species as to their redox state and the next binding atom. The community using mass spectrometry (MS), however, would like to determine the entire molecular forms of an element. Needless to say, the MS methods also fail sometimes to determine the correct molecular structure, especially in the case of stereochemistry. This can be solved only by purification and preconcentration followed by use of NMR methods.

The speciation community arose mainly through the rise of atomic spectrometry and in particular elemental MS; that is, inductively coupled plasma MS (ICPMS). The ionization source of an argon plasma is so powerful that that no species information on the introduced molecule remains. Although it sounds like an oxymoron, this is exactly what makes ICPMS the ideal detection method for speciation analysis. The detector, however, needs to be coupled to something which is selective to the nature of the molecule rather than the elements in it. Since the early 1990s, classical methods for separating molecules such as chromatography [high-performance liquid chromatography (HPLC) and gas chromatography] have routinely been coupled with ICPMS, which acts as an element-specific detection method [3, 4]. Electrophoretic methods (capillary electrophoresis and gel electrophoresis) [5, 6] for the separation of rather unstable and/or high molecular weight species and flow fractionation methods (asymmetric flow field-flow fractionation) for the determination of nanoparticles [7] followed. Because of the elution behaviour, which is linked to the molecular properties, the individual elemental species can be determined by element-specific detection, and from the retention time and comparison with standards, their molecular nature can be revealed. This has been used for a decade as the sole identification method even for elemental species which had not been identified in nature before [8]. These identifications especially for species of the elements selenium and arsenic were mostly tentative and advanced the field to show the diversity of molecular forms occurring in biological systems. For the true identification of novel elemental species, molecular MS—that is, electrospray ionization MS (ESI-MS)—is needed as well. This technique can directly determine the molecular formulae by the accurate mass of the protonated molecular species, and structural information can be derived from the fragmentation pattern of the molecule itself (if it fragments). Full structures of novel compounds can often be deduced, although again here the information can be tentative with regard to the stereochemistry. The location of double bonds in lipids, for example, can be revealed only by NMR methods or from crystal structures by means of X-ray diffraction. Those methods, however, need pure compounds, which are often impossible to obtain when the elemental species in question occurs only in trace or ultra-trace concentration in a complex matrix.

However, ESI-MS by itself has not had the impact ICPMS had in the area of speciation analysis, although the technique can be more sensitive than ICPMS. The reasons are firstly that for the quantification of elemental species, species-specific standards are needed because of the ionization of the compounds being compound specific. Secondly, the signal intensity of a compound can dramatically be reduced when an easy-to-ionize compound is co-eluting with the compound in question. The latter can be addressed by separation of the compound of interest from the matrix by use of multidimensional chromatographic systems in which at least two or three different separation modes are combined [9] or by use of standard addition for quantification. Thirdly, non-target analysis of elemental species using solely molecular MS is often difficult, because compounds containing heteroatoms are not necessarily easy to identify amongst the other molecules. The use of algorithms to search specifically for heteroatom-containing molecular species is still difficult and near impossible when the element in question is a monoisotopic one. There is often a lack of algorithms which could be used to mine the enormous amount of data generated by ESI-MS methods, especially if the element in question is monoisotopic and the isotopic pattern cannot be used. However if a large mass defect exists (e.g. -0.07840 Da for As), this can be used to identify molecules which contain this element, as demonstrated for arsenic when high-resolution ESI-MS is used [10]. However if the mass defect is small and the number of heteroatoms in a molecule can be variable, then a useful algorithm cannot be written [11]. Hence, species identification in a complex matrix such as environmental and biological samples is often difficult because of the occurrence of hundreds of other organic molecules.

In contrast to ESI-MS, detection of heteroatom-containing compounds by ICPMS is easy, and that is why ICPMS became a very useful tool for non-target analysis of elements. It is particularly useful when combined with ESI-MS. But not only can ICPMS help find the “needle in the haystack” in the big data sets from ESI-MS, it can also be used for mass balance approaches because of the compound-independent element-specific response, which makes the quantification of novel compounds possible, often without direct knowledge of the molecular structure. It should be mentioned, however, that when a solvent gradient is used especially with organic solvents such as methanol, the response factor of the element can change during the chromatographic run and needs to be corrected as explained in more detail by Amayo et al. [12]. All these attributes of ICPMS made its popularity rise dramatically in the biological and environmental sciences, and ICPMS has been the motor of knowledge creation in the environmental chemistry and biochemistry of selenium and arsenic respectively.

**Fig. 1:**
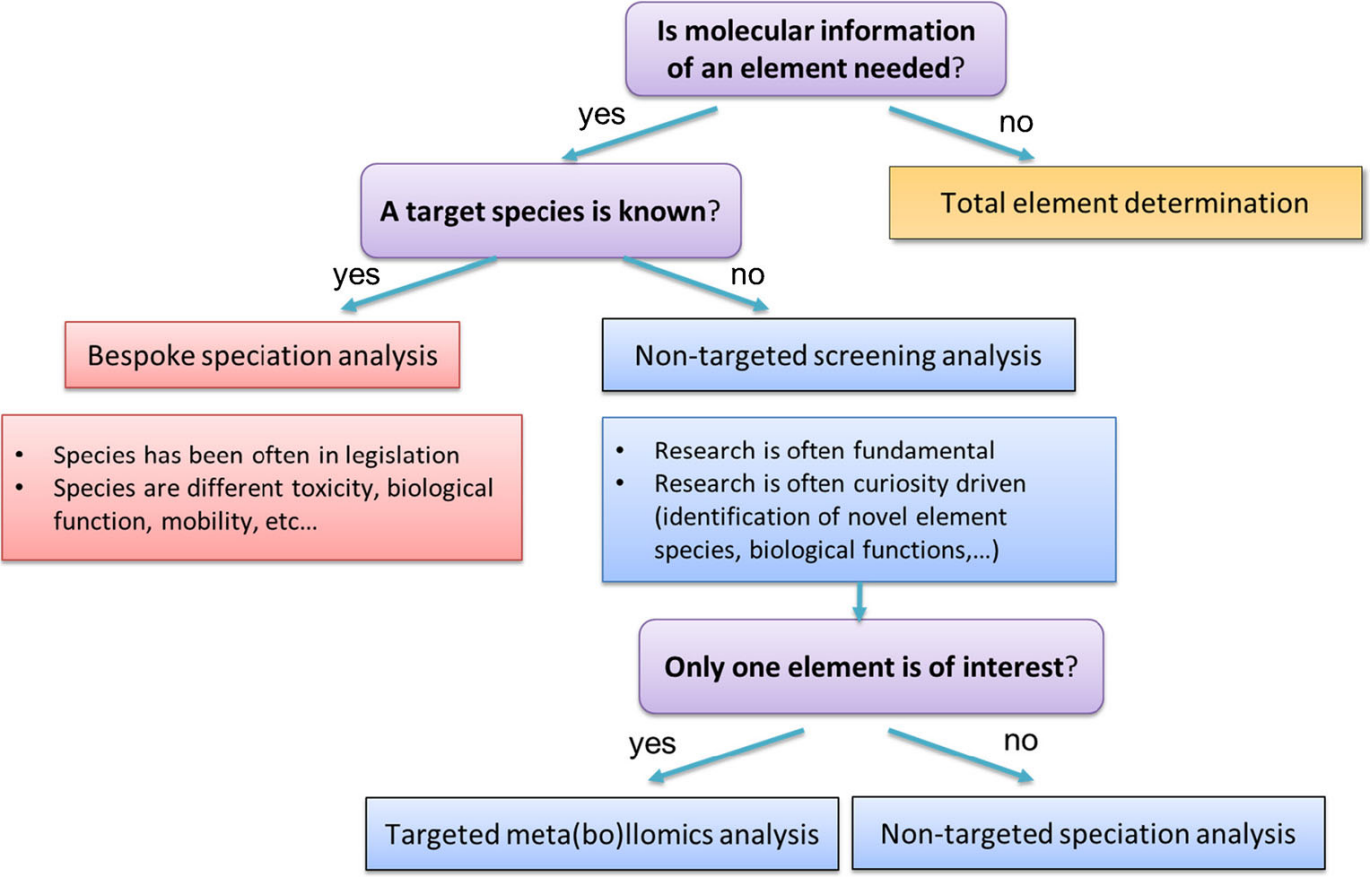
Characterization of different speciation studies according to the focus of the study. Choosing only one species of interest is a bespoke speciation analysis, whereas choosing one element for non-targeted analysis is a targeted meta(bo)llomics analysis. Non-targeted speciation analysis can also be described as a full metallomics analysis when all elements and all species are targeted

Examples of ICPMS use in speciation analysis can be divided into two main areas (Fig 1):

1. Bespoke methods for robust quantification of target elemental analytes
2. Non-targeted screening methods for identification of novel chemistry in biological and environmental sciences

## Bespoke methods for targeted speciation studies (status quo and outlook)

This area of speciation has been the focus of significant method developments. Species-specific isotope dilution methods have been described for tributyltin [13], methylmercury [14] and hexavalent chromium [15]. All these metal species have been identified as toxic compounds, and monitoring of these compounds in different samples such as sediments, organisms or drinking water is required by law. Therefore, absolute methods using species-specific isotope dilution methods have been developed to identify and also validate sample preparation approaches for these target species, and they give highly accurate and precise data [16]. This concept has only recently been extended to specific metalloproteins such as superoxide dismutase [17] and haemoglobin [18]. The accuracy and precision comes, however, at a price, since the methods are not cheap and they are complex and very slow. They are, however, vital in the validation of cheaper and faster assays for those target species. We will see here a development in which HPLC–ICPMS will be replaced by other methods.

For arsenic, the situation is more advanced. Speciation of arsenic is now integrated in legislation [19]: a maximal contaminant level for inorganic arsenic in rice was recommended by WHO/UN FAO and then subsequently implemented into EU law in January 2016. Hence, all rice and rice products need to be speciated for arsenic. The recommended method is HPLC–ICPMS. However, since small commercial laboratories do not necessarily have a coupled system dedicated to speciation analysis, there is a trend amongst analytical chemists to develop cheaper dedicated methods for these target species, such as solid-phase extraction coupled with hydride generation atomic absorption spectrometry [20], or selective hydride generation without chromatography coupled with ICPMS [21] or with atomic fluorescence spectroscopy [22]. Even a field-deployable method for screening has been developed for inorganic arsenic in rice [23] and seaweed [24].

Similar developments can also be seen for methylmercury, and these developments can, with the aid of preconcentration methods, easily be used for accurate determination of methylmercury at background-level (low parts per quadrillion) concentrations in HPLC–cyclic voltammetry–atomic fluorescence spectroscopy [25]. This is a trend which will continue, and the ICPMS detector will be replaced by cheaper, more specific detectors for routine or screening analysis; however, HPLC–ICPMS or gas chromatography–ICPMS will always be used as the gold standard for target analytes.

## Non-targeted speciation analysis (status quo and outlook)

When environmental and biological processes of a certain element rather than an established target species are the focus of a specific study, then it is important to understand how the different molecular forms of the element interchange in the system studied. Hence, it is necessary to identify all the different molecular forms of this one element in the sample. This means that we need a non-targeted speciation analysis which looks at all elemental species of the element studied. This type of non-targeted analysis is, however, more focussed than the classical non-targeted studies in metabolomics in which the information from the entire mass spectrum is used and only chemometric methods can unravel the importance of individual molecular species. Therefore, we should label these as selective non-target analysis, another oxymoron, but we feel that this describes it well, since the selectivity comes from looking at the molecules which contain one specific element only.

**Fig. 2:**
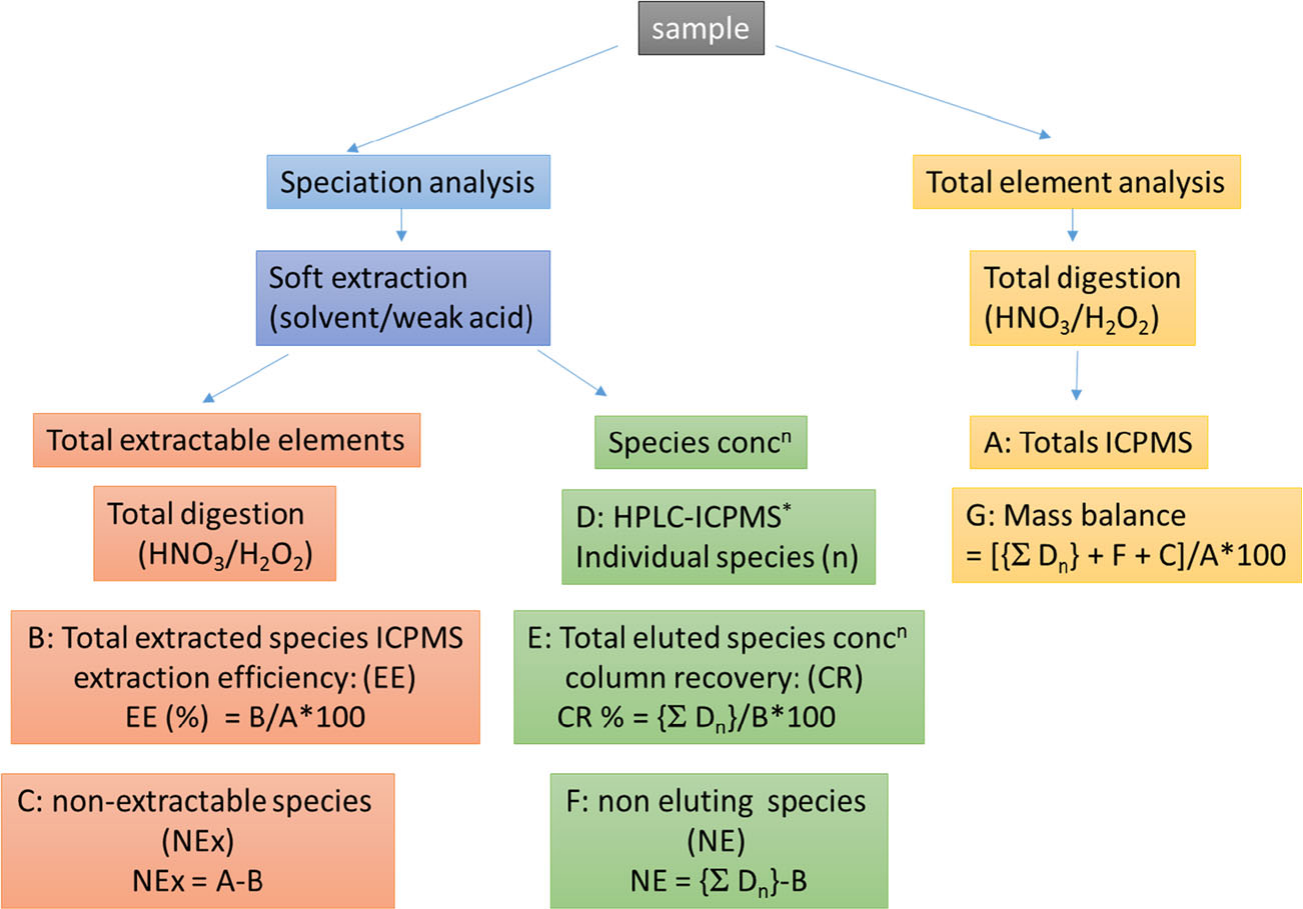
Quantification steps for non-targeted speciation analysis using high-performance liquid chromatography (*HPLC*)–inductively coupled plasma mass spectrometry (*ICPMS*). *A* is the total element concentration measured after full digestion with ICPMS; *B* is the total element concentration in the extract which conserves the speciation measured by ICPMS and then related to *A* to give the extraction efficiency; *C* is amount of the element which could not been extracted by extraction under *B*; *D* is all the concentrations of all individual elemental species for all *n* species identified by HPLC–ICPMS; *E* is the column recovery in percent; *F* is the amount of the element which is not eluted from the HPLC column; *G* is the overall mass balance

The non-targeted analysis of elemental species using mainly HPLC–ICPMS can not only identify the wealth of different molecular forms of an element but can also be used for mass balance approaches (Fig. 2), since the ICPMS detector shows a compound-independent response. The only exception occurs when compounds have different volatility, so the transport into the plasma can generate different analyte flows and therefore induces differences in the response. Hence, ICPMS can be used directly for the evaluation of how effective extraction methods are with regard to the element, rather than a specific target species, and whether the column recovery is quantitative when chromatography is used for the separation of elemental species. This was an invaluable tool for identification of “hidden” element species which either cannot be easily extracted or tend to stick on the column. This asset of ICPMS revealed the presence of a series of novel arsenic species, such as the occurrence of organothioarsenicals [26, 27] and new classes of arsenolipids [28, 29].

### Application: arsenic and selenium

When the redox chemistry and the organic chemistry of an element are as diverse as they are for selenium or arsenic, where hundreds of different molecular forms of these elements are known to occur in biota, then the simultaneous use of ICPMS and ESI-MS for a chromatographic separation is vital. Not only can the species be quantified (ICPMS), it can also at the same time be identified with use of use the ESI-MS data at the time the elemental species is eluted from the column according to the ICPMS signal. With use of this concept, a whole series of unstable arsenic, mercury and selenium phytochelatin or glutathione species have been identified which seem to be important for the transport mechanisms of these elements in plants [30, 31].

Predominantly, main-group elements which form a stable carbon bond have been studied with this approach, because the elemental species are relativity stable and can be separated by liquid chromatography. Non-targeted speciation studies of arsenic, selenium and in part mercury have dominated the literature in the past decade simply because there is a plethora of possible species and the analytical method used (here HPLC–ICPMS) can in general guarantee their stability [32].

### Application: transition metals

Transition elements only rarely form stable carbon bonds (e.g. cobalamin). These elements are, however, key essential elements in larger molecular compounds such as metalloproteins. The stabilities of the element–protein bonds often depend on the ternary structure of the protein, which means that their stability during sample preparation and separation is often compromised because of the denaturating non-cytosolic conditions used for extraction/cleaning or preconcentration steps. Additionally, complexation of metals by small organic molecules is important for metal trafficking and homeostasis in cells. However, that knowledge is obtained from molecular biology methods and crystal structures rather than from the use of HPLC–ICPMS.

The instability of these transition element complexes can, however, be overcome, as a recent study by Ghssein et al. [33] on nickel, iron, zinc and copper metabolites has shown. Here, the biosynthesis of a broad spectrum of metallophores was determined. These non-targeted methods can not only be focussed on one or a few elements but can also be extended to almost the entire element spectrum, as has been demonstrated by looking at the entire cytosolic metallome of a bacterium recently, in a truly metallomics approach [34]. Especially in this area of research we expect increased activity, especially orchestrated by biologists valuing ICPMS. Those non-targeted studies revealed novel metal species, and have been starting points for new scientific approaches.

There is, however, a warning sign for non-targeted speciation analysis, and that is the possibility of analytical artefact formation. Since no information is available on the elemental species in the sample, it is impossible to validate analytical results with the usual approaches, such as use of isotopically labelled elemental species or other orthogonal methods such as X-ray absorption near-edge spectroscopy or extended X-ray absorption fine structure spectroscopy. Therefore, species integrity cannot be guaranteed nor can it be known if the detected species are formed in situ during the sample preparation process.

### Application: non-metals

Some elements have rarely been studied by non-targeted elemental analysis despite their forming a large number of biological important compounds [35]. Among these are sulfur and the halogens, maybe with the exception of iodine. The reason for this is the traditionally low selectivity and sensitivity of ICPMS for those elements. Whereas sulfur is heavily interfered with by polyatomic oxygen species, and bromine is heavily interfered with by argides, chlorine and fluorine are not efficiently ionized in an argon plasma because of their extremely high ionization potentials. Although sulfur has been monitored to support the determination of thioarsenicals [36] or cysteine-bound elements such as selenium [37], selective non-targeted analysis for sulfur has not been reported. However, with the help of different ICPMS technologies such as high-resolution ICPMS [29] or ICPMS–MS [38] or simply the use of ultrasonic nebulizers with desolvation [39], the detection limits can be lowered to a few micrograms of sulfur per litre per eluted species. Bromine speciation has been used for targeted analysis of flame-retardant polybrominated diphenyl ether, but non-targeted analysis of brominated organic compound is missing, especially for natural products [40]. The authors believe that in particular marine organisms such as ascidians or seaweed may contain a large number of brominated compounds still waiting to be identified [41]. Additionally, brominated drugs have been studied by HPLC–ICPMS–MS in a non-targeted analysis approach [42, 43]. However, there seems to be no direct driving force for the study of the wealth of natural organobromine compounds, although the detection limits of ICPMS for bromine (below micrograms of Br per litre) are comparable to or even superior to those of other, cheaper but less selective methods such as electron-capture detection for volatile bromine compounds.

Most chlorinated pesticides are volatile and are analysed with relatively selective detectors such as an electron-capture detector, but the use of electron-capture detection for non-targeted analysis is limited to volatile compounds. Chlorinated compounds can, in principle, be identified from ESI-MS data and their isotopic pattern, but cannot be quantified without species-specific standards. Although chlorine is not efficiently ionized in the argon plasma (ionization potential 13.0 eV) of the ICPMS system, elimination of the polyatomic interfering ions of *m*/*z* 35 and 37 with use of ICPMS–MS and hydrogen as the reaction gas can result in sub-part-per-million detection limits: for example, chlorine was measured as H_2_Cl^+^ [44]. Schwan et al. [44] separated and detected inorganic chlorine species (chlorite and chlorate) in the blood plasma of human volunteers. In this area, the authors expect that ICPMS will be used in the future for non-targeted analysis to identify unexpected or even novel chlorinated compounds in biological and environmental samples.

### Special case: fluorine

The situation for fluorine speciation seems, however, to be different. There are more than 2000 organofluorine compounds, mainly perfluorinated, registered by the chemical industry because of their interesting properties such as hydrophobicity and lipophobicity. Since these compounds are not biodegradable, the most prominent compound, perfluorooctanesulfonic acid, has been banned by the Stockholm Convention for further use. Hence, the analytical community developed targeted analysis for 10–15 perfluorinated compounds using isotopically labelled standards with HPLC–ESI-MS detection [45]. Although perfluorooctanesulfonic acid was banned more than a decade ago, 50–100 precursor perfluorinated compounds may exist, but standards do not exist for all of them [46]. This, however, demonstrates the problem with ESI-MS as a selective non-targeted analytical system, since the data cannot be mined successfully by mathematical algorithms to identify which molecules contain a given number of fluorine atoms. Here, a real necessity is the availability of a selective non-targeted analysis for organofluorine compounds in environmental and biological samples based on the element. There is a gap in the toolbox of the analytical chemist, since not even ICPMS can be used as a fluorine-specific detection method directly because of the high ionization potential (17.4 eV) in comparison with argon (15.7 eV); hence, there is insignificant production of F^+^. Screening of standard compounds by high-resolution ICPMS has been attempted, resulting in detection limits at the high parts per million level. These detection limits are, however, not low enough to measure background levels of perfluorinated compounds in environmental and biological samples. We have investigated the formation of polyatomic fluorine ions (such as BaF^+^) in the plasma, which can be used for the indirect detection of fluorine by ICPMS–MS and can be coupled with HPLC for fluorine speciation [47]. Organofluorine compounds are separated by HPLC, and once the compound enters the plasma, it is atomized. The generated F^-^ and/or atomic F^0^ form polyatomic ions such as BaF^+^, which can be detected in trace concentrations (micrograms of F per litre range). This method has not yet been applied to environmental samples for speciation, but for total fluorine it looks promising [48]. In this area we will see increased activities, and we are sure that this will influence how organofluorine analysis is performed in the future. This specific non-targeted analysis will be able to identify so far hidden organofluorine compounds not only in the area of perfluorinated compounds but also in pharmaceutical applications—20% of all new pharmaceuticals are fluorinated [49]. However, future studies need to show how robust the detection of fluorine is by use of polyatomic ions. The authors hope that this increased activity from an area which is not traditionally ICPMS terrain will encourage instrument manufacturers either to develop dedicated fluorine detectors or to produce again a negative-ion ICPMS instrument.

## Outlook

There is evidence that study of the elemental species which are studied by the analytical community is driven not necessarily by need but more often by instrumental capabilities. Many articles about arsenic speciation exist and are published on a weekly basis without driving the field of arsenic speciation forward, whereas there is a real need to understand ligand–metal interactions, especially for essential elements. But those elemental species often remain elusive because of the lack of stability outside cytosolic conditions, whereas an entire community of environmental scientists interested in perfluorinated compounds is looking for a selective non-targeted analysis method for fluorinated compounds. We expect that ICPMS systems, in the long term, will be replaced by dedicated instruments for target species, whereas fundamental studies and applications are expected for non-metal non-targeted analysis, with a focus on sulfur, chlorine and fluorine speciation.
